# Hepatitis E virus prevalence among blood donors in Dali, China

**DOI:** 10.1186/s12985-021-01607-y

**Published:** 2021-07-07

**Authors:** Ping Fu, Baochai Lin, Bingting Wu, Ling Ke, Tianfu Yang, Yue’e Du, Lishan Cheng, Zhou Li, Tiancheng Li, Yu Liu

**Affiliations:** 1 Transfusion Medicine Research Center, The Institute of Blood Transfusion, Chinese Academy of Medical Sciences and Peking Union Medical College, Chengdu, 610052 China; 2 Dali Blood Center, Dali, Yunnan China; 3 Department of Virology II, National Institute of Infectious Diseases, Gakuen 4-7-1, Musashi-murayama, Tokyo 208-0011 Japan

**Keywords:** HEV, Prevalence, Blood donor, China

## Abstract

**Background:**

Hepatitis E virus (HEV) is a nonenveloped RNA virus causing hepatitis E worldwide. The increase in transfusion-transmitted cases of HEV infections from asymptomatic blood donors causing serious illnesses among immunosuppressed recipients has been reported in the past few years. China is one of the most prevalent regions of HEV; as a result, it is important to evaluate the risk of transfusion-transmitted HEV.

**Methods:**

A total of 1864 serum samples (including demographic characteristics) from blood donors were randomly collected from February to March 2018 in Dali city. Anti-HEV IgG, IgM and IgA antibodies and HEV antigen were examined by enzyme-linked immunosorbent assay (ELISA). HEV RNA was detected by real-time PCR. Multivariable logistic regression modelling was used to examine the risk factors associated with HEV prevalence.

**Results:**

Overall, the positive rates of anti-HEV IgG, IgM, and IgA antibodies were 13.36% (249/1864), 1.13% (21/1864), and 1.82% (34/1864), respectively. However, none of the 1864 serum samples were HEV antigen positive or HEV RNA positive. Females (16.69%) had a significantly higher HEV seroprevalence than males (13.04%) (odds ratio [OR] 1.34 [95% CI, 1.02–1.75]). Bai (18.85%) donors had a significantly higher HEV seroprevalence than Han (12.21%) blood donors (odds ratio [OR], 1.65 [95% CI, 1.24–2.19] for Bai).

**Conclusions:**

HEV showed a seroprevalence among blood donors in Yunnan Province, some of which were even recent infections, indicating a threat to the safety of blood transfusions. Whether to formulate a strategy for HEV screening in blood centres needs further research.

**Supplementary Information:**

The online version contains supplementary material available at 10.1186/s12985-021-01607-y.

## Background

Hepatitis E virus (HEV) is an enteric RNA virus causing hepatitis E infection worldwide. Similar to hepatitis A virus infection, only a portion of infected patients experience HEV-related symptoms [1].

In July 2019, the World Health Organization (WHO) reported that approximately 20 million people are infected by HEV each year, and 44,000 people died in 2015. China is one of the highly prevalent regions of HEV with a seroprevalence from 0.01 to 48% [2]. A very large outbreak of hepatitis E was reported in the Xinjiang Uighur Autonomous Region during 1986–1988, causing 119,280 cases and more than 700 deaths [3].

HEV is usually transmitted through drinking water and food contaminated by HEV infectors [3]. Since water supplies and sanitary infrastructures have improved, animals have become the major source of human HEV infection [4]. Moreover, an increase in transfusion-transmitted cases of HEV infections from asymptomatic blood donors causing serious illnesses among immunosuppressed recipients has been reported in the past few years [5–11]. To protect the patient from transfusion-acquired HEV infection, blood components are analysed by HEV screening before they are provided to at-risk patients in the United Kingdom [12] and all blood recipients in Switzerland [13].

Dali is a traffic stronghold and famous tourist city in western Yunnan, China, with a complex population and epidemic background of infectious diseases. Because people in Dali eat raw or undercooked food and are in contact with animals frequently, along with the relatively low living standard of residents, there is a high HEV infection rate in this region [14]. This study aims to provide an estimation of HEV prevalence among blood donors in Yunnan, China, to evaluate the risk of HEV transmission by blood donation and to identify the risk factors associated with HEV infection.

## Methods

### Sample collection

This study was approved by the Ethics Committee of the Institute of Blood Transfusion, Chinese Academy of Medical Sciences (IBT). A total of 1864 donation blood samples were obtained randomly from February to March 2018 in Dali, China. All donor samples were tested for alanine aminotransferase (ALT), HBsAg, anti-HCV, anti-HIV-1/2, and syphilis during routine donor screening. Questionnaires about demographic and donation characteristics were voluntarily completed by the donors, including sex, age, race/ethnicity, education, occupation, donation times, and history of consumption of raw food such as beef, mutton or milk (Additional file 1: Fig. S1). Test samples were stored in − 80 °C freezers at the blood centre until they were shipped in batches to IBT in dry ice-type environments.

### Detection of anti HEV-IgG, Anti HEV-IgM and HEV IgA antibodies

ELISAs for the detection of anti-HEV antibodies were established by using HEV-like particles (HEV-LPs) as the antigen, which was produced by recombinant baculoviruses [15]. Microplates (96-well) were coated with 200 ng/well HEV-LPs with 0.05 M carbonate-bicarbonate buffer (pH 9.6) at 4 °C overnight and blocked with 100 µl 10% nonfat milk (Sigma, China) at 37 °C for 2 h. After washing with PBS-T three times, 100 µL of 1:200 diluted plasma samples were added and incubated at 37 °C for 1 h. After five washes, each well was supplemented with 100 µl of horseradish peroxidase (HRP)-conjugated goat anti-human IgG (1:20,000 diluted) (Cappel, Durham, NC) or IgM (1:10,000 diluted) (Bethyl, USA) or IgA (1:10,000 diluted) (Bethyl, USA) antibody and incubated at 37 °C for 1 h. Then, the plates were washed four times with PBS-T, 100 µl of TMB/H_2_O_2_ (Beyotime, Shanghai, China) was added, and incubated in a dark room for 15 min at room temperature. The enzymatic reaction was stopped with 50 µl 0.3 M sulfuric acid, and the optical density (OD) values were measured at 450 nm. The cut-off value was determined based on the mean OD_450nm_ value of the negative control (NC) by the following formula: Cut-off = 2.1 * NC_mean_. Values of OD_450nm_ < Cut-off indicated a negative sample, and ≥ Cut-off indicated a positive sample. Ten plasma samples collected from donors without a history of HEV infection were used as a negative control.

### Detection of the capsid antigens of HEV

HEV antigens were detected by a two-step incubation antibody-based sandwich ELISA kit (Wantai, Beijing, China). The procedure was carried out according to the manufacturer’s instructions. The cut-off values of the assay were statistically established as the mean optical density value of negative controls at a 450-nm optical wavelength plus 0.12.

### HEV-RNA detection

Nucleic acids were extracted from 200 µl of each sample using the Magen virus RNA kit (Shanghai, China) according to the manufacturer’s instructions. HEV RNA detection was accomplished by TaqMan® real-time fluorescence reverse transcription-polymerase chain reaction (RT-PCR). After extraction of viral RNA from 200 µl of serum by a viral DNA/RNA mini kit (Magen, Shanghai, China), 30 µl of diethylpyrocarbonate (DEPC)-treated water was added. For TaqMan® RT-PCR, the 20 µl reaction contained 4 µl of 5 × QuantiTect Probe RT-PCR kit Master Mix (Magen, Shanghai, China), 0.2 µl of enzyme, 10 µl of RNA, and primers and probe at concentrations of 250 and 100 nM, respectively. Primers and probes have been widely used in many reports for four major HEV genotypes based on the multiple sequence alignments of 27 sequences of the ORF3 region [16]. PCR was performed on a sequence detection system platform (ABI Prism 7500, Applied Biosystems) as follows: reverse transcription was carried out at 50 °C for 5 min, followed by denaturation at 95 °C, then 40 cycles of denaturation at 95 °C for 10 s and annealing and extension at 60 °C for 30 s. The TaqMan® assay detected as few as 5 genome equivalent (GE) copies of HEV plasmid DNA. The sequence of the plasmid (pUCm-T vector, sangon) is shown in Additional file 2.

### Statistical analysis

The chi-square test was used to assess the anti-HEV IgG, IgM, and IgA positive rates by donor demographics, donation characteristics, and history of consumption of raw food. The multivariable logistic regression model was then fit to examine factors associated with anti-HEV positivity. The traditional principle was used to define HEV seroprevalence: for results of anti-HEV IgG in combination with IgM, whenever anti-HEV IgG or IgM was positive. The multivariable logistic regression model was fit to examine factors associated with HEV seroprevalence. All statistical analyses were performed using the statistical software package SPSS 23 (SPSS Inc., Chicago, IL). A *p *value of 0.05 or less was considered to indicate significant differences.

## Results

### HEV prevalence

Among the 1864 blood samples collected from donors in the Dali Blood Center, the positive rates for anti-HEV IgG, IgM, and IgA among Dali donors were 13.35% (249/1864), 1.12% (21/1864), and 1.82% (34/1864), respectively (Table 1). When donors had any reactive results of anti-HEV IgG or IgM, they were defined as HEV seropositive. The HEV seroprevalence was 14.22% (265/1864) (as listed in Table 1).

**Table 1:** HEV serologic test results among 1864 blood samples in Dali

HEV biomarkers	Reactive number	Reactive rate (%)	95% confidence interval (%)
Anti-HEV IgG	249	13.36	11.81% ~ 14.90
Anti-HEV IgM	21	1.13	0.65% ~ 1.61
Anti-HEV IgA	34	1.82	1.22% ~ 2.43
HEV seroprevalence	265	14.22	12.78% ~ 15.97
Anti-HEV IgG/IgM/IgA	278	14.91	13.30% ~ 16.53

*HEV seroprevalence* has any reactive result of anti-HEV IgG and anti-HEV IgM, *Anti-HEV IgG/IgM/IgA* has any reactive result of anti-HEV IgG, anti-HEV IgM and anti-HEV IgA

Among the 1864 donor samples, none of them were HEV antigen positive or HEV RNA positive.

### Risk factors

Seven samples were identified as unqualified with ALT levels higher than 50 U/L, which is the blood screening limit in China. The positive rate of anti-HEV IgG antibody in donors with ALT > 50 U/L was higher than that in other donors, but no significant difference was observed (*P* = 0.238) (Table 2).

**Table 2:** HEV results of routine screening of unqualified or grey zone samples

	Value	Anti-HEV IgG	Anti-HEV IgM	Anti-HEV IgA	HEV antigen	HEV RNA
ALT	52 U/L	−	−	−	−	−
	55 U/L	+	−	−	−	−
	69 U/L	−	−	−	−	−
	51 U/L	−	−	−	−	−
	51 U/L	−	−	−	−	−
	51 U/L	+	−	−	−	−
	52 U/L	−	−	−	−	−

+: reactive; −: nonreactive

**Fig. 1:**
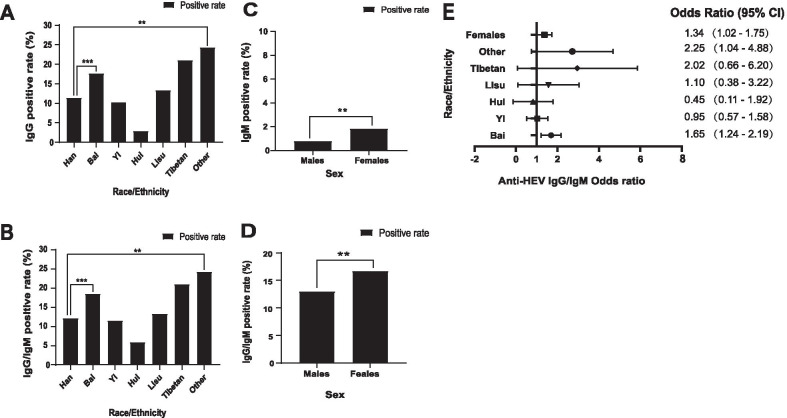
Comparison of the difference in the HEV biomarker positivity rate among different nationalities and sexes. **a** Anti-HEV IgG positivity rate in different races. **b** HEV seropositivity rate in different races. **c** Anti-HEV IgM positivity rate in different sexes. **d** HEV seropositivity rate in different sexes. **e** Multivariable logistic regression analysis of HEV seroprevalence (anti-HEV IgG or IgM) in different sexes and ethnic minorities

In the chi-square test and multivariable logistic regression analysis of HEV seroprevalence, females (16.69%) had a significantly higher seroprevalence than males (13.04%) (*P* < 0.05; odds ratio [OR]: 1.34 [95% CI, 1.02–1.75]). Other ethnic minority (24.32%) and Bai (18.85%) donors had a significantly higher seroprevalence than Han (12.21%) blood donors (*P* < 0.05, odds ratio [OR], 2.25 [95% CI, 1.04–4.88] for other ethnic minorities; *P* < 0.001, 1.65 [95% CI, 1.24–2.19] for Bai) (Fig. 1) (Additional file 3: Table S1 and Additional file 4: Table S2.)

**Fig. 2:**
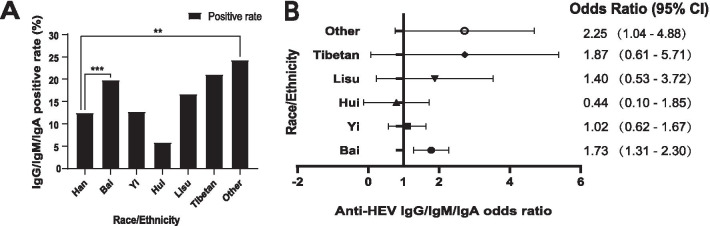
Comparison of the difference in anti-HEV IgG/IgM/IgA among different nationalities and sexes. **a** Anti-HEV IgG/IgM/IgA in different races. **b** Multivariable logistic regression analysis of anti-HEV IgG/IgM/IgA in different sexes and ethnic minorities

Similar chi-square and multivariable analysis results were also found for anti-HEV IgG/IgM/IgA when compared to Han blood donors (OR, 2.25 [95% CI, 1.04–4.88] for other ethnic minorities, 1.73 [95% CI, 1.31–2.30] for Bai) (Fig. 2*.*) (Additional file 3: Table S1 and Additional file 4: Table S2). No statistically significant difference in anti-HEV IgG, anti-HEV IgM, anti-HEV IgA, HEV seroprevalence, or anti-HEV IgG/IgM/IgA was found by age, education, occupation, marital status, donation times, or diet history (Additional file 3: Table S1).

## Discussion

Yunnan, a highly endemic region for HEV as reported by the data centre of China public health science (http://www.phsciencedata.cn), showed a rapid increasing trend in hepatitis E incidence, ranging from 1.83 to 3.01% during 2014–2016 (Mean: 2.48 ± 0.60 per 100,000 person-years). An increasing number of blood transfusion-caused HEV cases have been reported in recent years, yet Chinese blood centres do not routinely perform screening testing for HEV [17, 18]. In this study, 14.22% of blood donors were HEV seroprevalence positive, showing that there might be a risk of blood transfusion transmission.

Although no direct evidence of transfusion transmission of HEV, HEV antigen or HEV nucleic acid was found in blood donors in this study, 1.13% of blood donors were anti-HEV IgM positive, and 1.84% of blood donors were anti-HEV IgA positive, which represented a recent infection and may create the risk of transfusion-transmitted HEV infection [19–22]. Existing studies have shown that the prevalence of HEV RNA among blood donors was 0.045% in France [23], 0.0371% in the Netherlands [24] and 0.03% in Spain [7]. Although none of the HEV RNA or HEV antigen-positive samples was detected in this study, this may be due to the limited sample size, in which only 1864 cases were involved, meaning that the prevalence of HEV RNA or antigen among blood donors in Dali is likely lower than 0.054%. Therefore, this does not mean that HEVs are not transfusion transmitted but means that the specific risk of transfusion-transmitted HEV infection by blood products in China remains unclear. It is suggested that scholars who study the risk of HEV transmission by blood donation should increase the sample size.

The risk factors for HEV infection in blood donors were also analysed, and it was found that females had a significantly higher anti-HEV IgG/IgM prevalence than males (*P* < 0.05) (OR, 1.34 [95% CI, 1.02–1.75] for females compared to males)(Fig. 1). This result contradicts other published literature [18, 25, 26]. We speculate that it may due to the Regional and dietary differences. In Dali, males work mainly in construction, catering or tourism, while females mainly do housework or farm work, like breeding and planting of rice which are more likely to exposed to HEV infected water. It is believed that consumption of uncooked or undercooked infectious HEV-contaminated meat or milk could be a new zoonotic source that bears a high risk of transmission to humans [27].

Besides, we also found that Bai nationality had a significantly higher anti-HEV IgG/IgM prevalence than Han nationality (*P* < 0.05). (1.65 [95% CI, 1.24–2.19] for Bai compared to Han) (Fig. 1). Dali is the origin and main settlement of Bai nationality with the custom of eating raw meat. It is believed that consumption of uncooked or undercooked infectious HEV contaminated meat or milk could be the new zoonotic source and bear a high transition of to human [27, 28]. However, no significant difference in HEV seroprevalence estimation among blood donors was found by the consumption of uncooked milk or meat (beef or mutton) in this study. The reason might be that our sample size was relatively small, and raw pork was not included in the scope of the study. What’s more, China is highly diverse in human distribution and culture, which means almost all of the 56 ethnic groups in China have their own ethnic communities. These ethnic groups have their own characteristics in terms of origin, genetic phenotype [29] as well as the types and incidence of genetic diseases or the susceptibility to other diseases [30]. We speculated that the differences in genetic information among ethnic groups may lead to variations in HEV susceptibility and further affect the prevalent trend of HEV.

Further genetic and epidemiological surveys are essential to identify the reasons for these differences, including genomic analysis and information collection of raw pork consumption, untreated water contact and farming condition.

## Conclusions

Generally, HEV showed a seroprevalence among blood donors in Yunnan Province, some of which were even recent infections, indicating a threat to the safety of blood transfusion. Whether to formulate a strategy for HEV screening in blood centres needs further research.

## Supplementary Information

**Additional file 1: Fig S1.** Demographic characteristics of the 1864 donors who completed the questionnaire.

**Additional file 2.** The sequence of HEV plasmid DNA.

**Additional file 3: Table S1.** HEV seroprevalence rates based on demographic and donation characteristics among Yunnan donors.

**Additional file 4: Table S2.** Multivariable logistic regression analysis of HEV seroprevalence.

## Data Availability

The data analysed during the current study are available from the corresponding author on reasonable request.

## Abbreviations

HEV: hepatitis E virus
RNA: ribonucleic acid
DNA: deoxyribonucleic acid
WHO: World Health Organization
IBT: Institute of Blood Transfusion
ALT: alanine aminotransferase
HEV-LPs: HEV-like-particles
