# Supplementary material for: Hepatitis E virus prevalence among blood donors in Dali, China
Source: Virol J. 2021 Jul 7;18:141. doi: 10.1186/s12985-021-01607-y (PMC8261953; doi:10.1186/s12985-021-01607-y)
Supplement: Supplementary file 1 — Additional file 1: Fig S1. Demographic characteristics of the 1864 donors who completed the questionnaire. [file 12985_2021_1607_MOESM1_ESM.pdf]

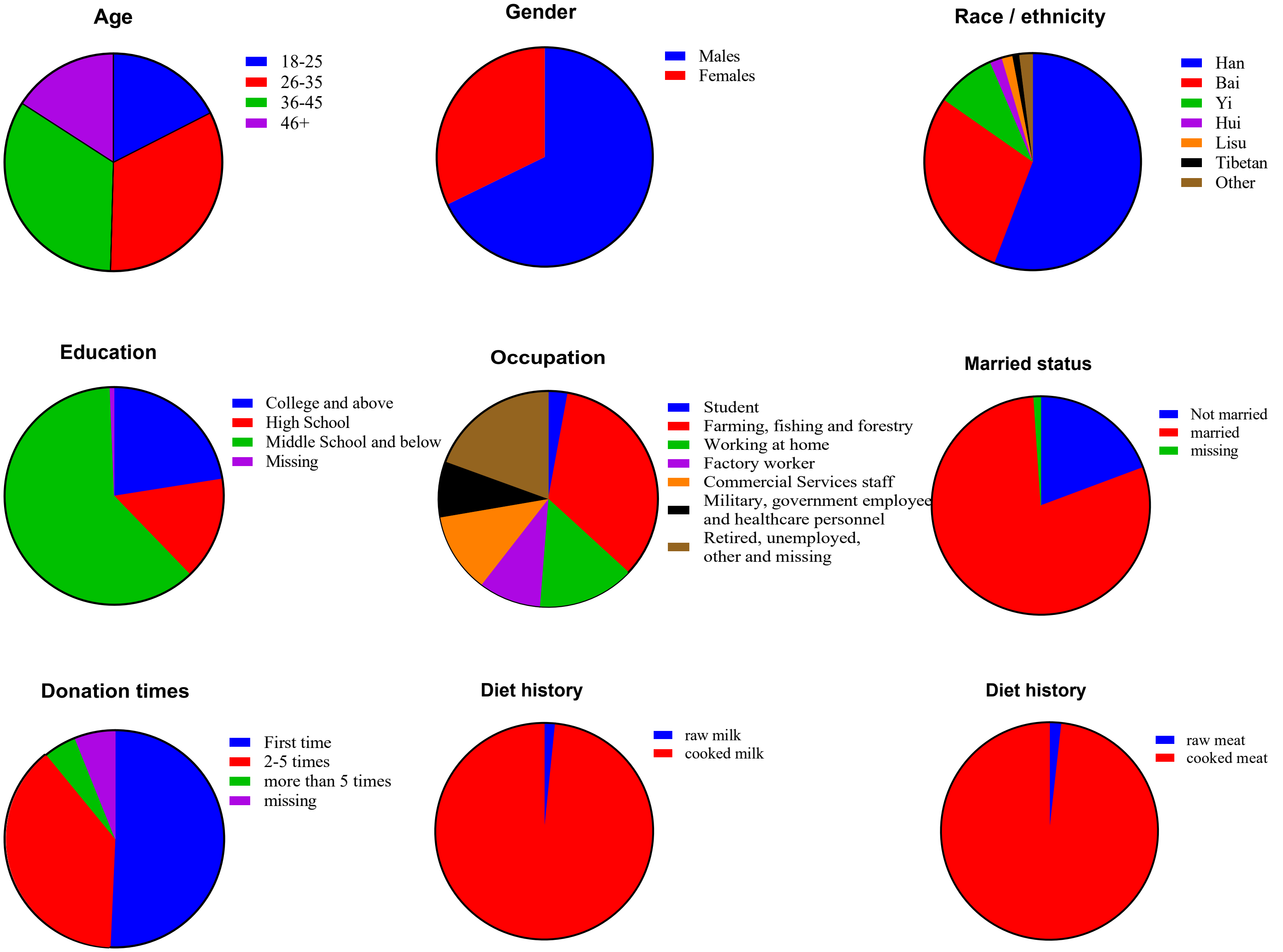

Age
18-25
26-35
36-45
46+
Gender
Males
Females
Race / ethnicity
Han
Bai
Yi
Hui
Lisu
Tibetan
Other
Education
College and above
High School
Middle School and below
Missing
Occupation
Student
Farming, fishing and forestry
Working at home
Factory worker
Commercial Services staff
Military, government employee and healthcare personnel
Retired, unemployed, other and missing
Married status
Not married
married
missing
Donation times
First time
2-5 times
more than 5 times
missing
Diet history
raw milk
cooked milk
Diet history
raw meat
cooked meat
