# Supplementary material for: Hepatitis E virus prevalence among blood donors in Dali, China
Source: Virol J. 2021 Jul 7;18:141. doi: 10.1186/s12985-021-01607-y (PMC8261953; doi:10.1186/s12985-021-01607-y)
Supplement: Supplementary file 2 — Additional file 2. The sequence of HEV plasmid DNA. [file 12985_2021_1607_MOESM2_ESM.docx]

The sequence of HEV plasmid DNA:

5’- GCAGACTATCGCTGATGGTAAGGCCCATTTTACAGAGACTGTTAAACCTGTGCTTGATCTTACAAATTCTATCGTACAGCGGATAGAATGAATAACATGTTTTGTGCATTGCCCATGGGGTCACCATGTGCCCTAGGGCTGTTCTGTTGCTGTTCTTCGTGCTTCTGCCTATGCTGCCCGCGCCACCGGCCGGCCAGCCGTCTGGCCGCCGTCGTGGGCGGCGCAGCGGCGGTACCGGCGGTGGTTTCTGGGGTGACAGGGTTGATTCTCAGCCCTTCGCCCTCCCCTATATTCATCCAACCAACCCCTTCGCCGCCGATGTCGTTTCACAATCCGGGGCTGGAGCTCGCCCTCGACAGCCGCCCCGCCCCCTTGGCTCCGCTTGGCGTGA-3’.
