# Supplementary material for: Hepatitis E virus prevalence among blood donors in Dali, China
Source: Virol J. 2021 Jul 7;18:141. doi: 10.1186/s12985-021-01607-y (PMC8261953; doi:10.1186/s12985-021-01607-y)
Supplement: Supplementary file 3 — Additional file 3: Table S1. HEV seroprevalence rates based on demographic and donation characteristics among Yunnan donors. [file 12985_2021_1607_MOESM3_ESM.docx]

**Table S1. HEV seroprevalence rates based on demographic and donation characteristics among Yunnan donors.**

| **Demographic and donation characteristics** | **Total Number of Donors** | **HEV IgG** | | **HEV IgM positive** | | **HEV IgA positive** | | **HEV seroprevalence**  **(anti-HEV IgG/IgM)** | | **HEV seroprevalence (anti-HEV IgG/IgM/IgA)** | |
| --- | --- | --- | --- | --- | --- | --- | --- | --- | --- | --- | --- |
|  |  | **Number of Reactive Donors (%)** | ***p-value*** | **Number of Reactive Donors (%)** | ***p-value*** | **Number of Reactive Donors (%)** | ***p-value*** | **Number of Reactive Donors (%)** | ***p-value*** | **Number of Reactive Donors (%)** | ***p-value*** |
| **Age (years old)** | | | | | | | | | | | |
| 18-25 | 326 | 34 (10.43) | 0.08 | 4 (1.23) | 0.11 | 4 (1.23) | 0.07 | 37 (11.35) | 0.15 | 37 (11.35) | 0.13 |
| 26-35 | 614 | 75 (12.21) |  | 11 (1.79) |  | 10 (1.63) |  | 84 (13.68) |  | 89 (14.50) |  |
| 36-45 | 628 | 100 (15.92) |  | 6 (0.96) |  | 9 (1.43) |  | 104 (16.56) |  | 107 (17.04) |  |
| 46+ | 296 | 40 (13.51) |  | 0 (0.00) |  | 11 (3.72) |  | 40 (13.51) |  | 45 (15.20) |  |
| **Sex** | | | | | | | | | | | |
| Males | 1,265 | 158 (12.49) | 0.11 | 10 (0.79) | **0.046** | 21 (1.66) | 0.44 | 165 (13.04) | **0.04** | 177 (13.99) | 0.10 |
| Females | 599 | 91 (15.19) |  | 11 (1.84) |  | 13 (2.17) |  | 100 (16.69) |  | 101 (16.86) |  |
| **Race/Ethnicity** | | | | | | | | | | | |
| Han | 1,040 | 119 (11.44) | **0.002** | 9 (0.87) | 0.51 | 15 (1.44) | 0.32 | 127 (12.21) | **0.005** | 130 (12.50) | **0.002** |
| Bai | 539 | 95 (17.63) |  | 7 (1.30) |  | 14 (2.60) |  | 100 (18.55) |  | 107 (19.85) |  |
| Yi | 165 | 17 (10.30) |  | 4 (2.42) |  | 2 (1.21) |  | 19 (11.52) |  | 21 (12.73) |  |
| Hui | 34 | 1 (2.94) |  | 1 (2.94) |  | 0 (0.00) |  | 2 (5.88) |  | 2 (5.88) |  |
| Lisu | 30 | 4 (13.33) |  | 0 (0.00) |  | 1 (3.33) |  | 4 (13.33) |  | 5 (16.67) |  |
| Tibetan | 19 | 4 (21.05) |  | 0 (0.00) |  | 0 (0.00) |  | 4 (21.05) |  | 4 (21.05) |  |
| Other | 37 | 9 (24.32) |  | 0 (0.00) |  | 2 (5.41) |  | 9 (24.32) |  | 9 (24.32) |  |
| **Education** | | | | | | | | | | | |
| College and above | 419 | 45 (10.74) | 0.21 | 6 (1.43) | 0.81 | 4 (0.95) | 0.06 | 51 (12.17) | 0.26 | 52 (12.41) | 0.13 |
| High School | 286 | 46 (16.08) |  | 4 (1.40) |  | 9 (3.15) |  | 50 (17.48) |  | 54 (18.88) |  |
| Middle School and below | 1147 | 156 (13.60) |  | 11 (0.96) |  | 20 (1.74) |  | 162 (14.12) |  | 170 (14.82) |  |
| Missing | 12 | 2 (16.67) |  | 0 (0.00) |  | 1 (8.33) |  | 2 (16.67) |  | 2 (16.67) |  |
| **Occupation** | | | | | | | | | | | |
| Student | 53 | 4 (7.55) | 0.79 | 1 (1.89) | 0.60 | 1 (1.89) | 0.51 | 5 (9.43) | 0.78 | 5 (9.43) | 0.80 |
| Farming, fishing and forestry | 635 | 86 (13.54) |  | 6 (0.94) |  | 14 (2.20) |  | 88 (13.86) |  | 98 (15.43) |  |
| Working at home | 267 | 41 (15.36) |  | 2 (0.75) |  | 4 (1.50) |  | 43 (16.10) |  | 43 (16.10) |  |
| Factory worker | 171 | 21 (12.28) |  | 3 (1.75) |  | 1 (0.58) |  | 24 (14.04) |  | 24 (14.04) |  |
| Commercial Services staff | 221 | 31 (14.03) |  | 5 (2.26) |  | 2 (0.90) |  | 36 (16.29) |  | 36 (16.29) |  |
| Military, government employee and healthcare personnel | 156 | 18 (11.54) |  | 1 (0.64) |  | 2 (1.28) |  | 19 (12.18) |  | 19 (12.18) |  |
| Retired, unemployed, other and missing | 361 | 48 (13.3) |  | 1 (0.28) |  | 10 (2.77) |  | 50 (13.85) |  | 53 (14.68) |  |
| **Married status** | | | | | | | | | | | |
| Not married | 360 | 36 (10.00) | 0.11 | 4 (4.11) | 0.89 | 5 (1.39) | 0.64 | 39 (10.83) | 0.12 | 40 (11.11) | 0.078 |
| married | 1,484 | 210 (14.15) |  | 17 (1.15) |  | 29 (1.95) |  | 223 (15.03) |  | 235 (15.84) |  |
| missing | 20 | 3 (15.00) |  | 0 (0.00) |  | 0 (0.00) |  | 3 (15.00) |  | 3 (15.00) |  |
| **Donation times** | | | | | | | | | | | |
| First time | 945 | 135 (14.29) | 0.44 | 12 (1.27) | 0.69 | 20 (2.12) | 0.79 | 144 (15.24) | 0.47 | 151 (15.98) | 0.50 |
| 2-5 times | 716 | 90 (12.57) |  | 8 (1.12) |  | 11 (1.54) |  | 96 (13.41) |  | 100 (13.99) |  |
| more than 5 times | 90 | 8 (8.89) |  | 1 (1.10) |  | 1 (1.10) |  | 9 (10.00) |  | 10 (10.99) |  |
| missing | 113 | 16 (14.16) |  | 0 (0.00) |  | 2 (1.77) |  | 16 (14.20) |  | 17 (15.04) |  |
| **Diet history** | | | | | | | | | | | |
| raw milk | 30 | 7 (23.33) | 0.18 | 0 (0.00) | 1 | 2 (6.67) | 0.19 | 7 (23.33) | 0.24 | 7 (23.33) | 0.30 |
| cooked milk | 1,834 | 242 (13.20) |  | 21 (1.15) |  | 32 (1.74) |  | 258 (14.07) |  | 271 (14.78) |  |
| **Diet history** | | | | | | | | | | | |
| raw meat | 33 | 5 (15.15) | 0.96 | 1 (3.03) | 0.83 | 0 (0.00) | 1 | 6 (18.18) | 0.68 | 6 (18.18) | 0.78 |
| cooked meat | 1,831 | 244 (13.33) |  | 20 (1.09) |  | 34 (1.82) |  | 259 (14.15) |  | 272 (14.86) |  |
| **Total** | 1864 | 249 (13.35) | / | 21 (1.12) | / | 34 (1.82) | / | 265 (14.22) |  | 278 (14.91) | / |
