# Supplementary material for: Hepatitis E virus prevalence among blood donors in Dali, China
Source: Virol J. 2021 Jul 7;18:141. doi: 10.1186/s12985-021-01607-y (PMC8261953; doi:10.1186/s12985-021-01607-y)
Supplement: Supplementary file 4 — Additional file 4: Table S2. Multivariable logistic regression analysis of HEV seroprevalence. [file 12985_2021_1607_MOESM4_ESM.docx]

**Table S2. Multivariable logistic regression analysis of HEV seroprevalence.**

| **Demographic and donation characteristics** | **Total Number of Donors** | **HEV seroprevalence (anti-HEV IgM/IgG)** | | | **Anti-HEV IgG/IgM/IgA** | | |
| --- | --- | --- | --- | --- | --- | --- | --- |
|  |  | **Number of Reactive Donors (%)** | ***p-value*** | **Odds Ratio (95% CI)** | **Number of Reactive Donors (%)** | ***p-value*** | **Odds Ratio (95% CI)** |
| **Sex** | | | | | | | |
| Males | 1,265 | 165 (13.04) | 0.04 | 1 | / | / | / |
| Females | 599 | 100 (16.69) |  | 1.34 (1.02, 1.75) | / |  | / |
| **Race/Ethnicity** | | | | | | | |
| Han | 1,040 | 127 (12.21) | 0.006 | 1 | 130 (12.50) | 0.002 | 1 |
| Bai | 539 | 100 (18.55) |  | 1.65 (1.24, 2.19) | 107 (19.85) |  | 1.73 (1.31, 2.30) |
| Yi | 165 | 19 (11.52) |  | 0.95 (0.57, 1.58) | 21 (12.73) |  | 1.02 (0.62, 1.67) |
| Hui | 34 | 2 (5.88) |  | 0.45 (0.11, 1.92) | 2 (5.88) |  | 0.44 (0.10, 1.85) |
| Lisu | 30 | 4 (13.33) |  | 1.10 (0.38, 3.22) | 5 (16.67) |  | 1.40 (0.53, 3.72) |
| Tibetan | 19 | 4 (21.05) |  | 2.02 (0.66, 6.20) | 4 (21.05) |  | 1.87 (0.61, 5.71) |
| Other | 37 | 9 (24.32) |  | 2.25 (1.04, 4.88) | 9 (24.32) |  | 2.25 (1.04, 4.88) |
| **Total** | 1864 | 265 (14.22) | / | / | 278 (14.91) | / | / |
